# When Painlevé–Gullstrand coordinates fail

**DOI:** 10.1140/epjc/s10052-020-8345-4

**Published:** 2020-08-25

**Authors:** Valerio Faraoni, Geneviève Vachon

**Affiliations:** Department of Physics and Astronomy, Bishop’s University, 2600 College Street, Sherbrooke, QC J1M 1Z7 Canada

## Abstract

Painlevé–Gullstrand coordinates, a very useful tool in spherical horizon thermodynamics, fail in anti-de Sitter space and in the inner region of Reissner–Nordström. We predict this breakdown to occur in any region containing negative Misner–Sharp–Hernandez quasilocal mass because of repulsive gravity stopping the motion of PG observers, which are in radial free fall with zero initial velocity. PG coordinates break down also in the static Einstein universe for completely different reasons. The more general Martel-Poisson family of charts, which normally has PG coordinates as a limit, is reported for static cosmologies (de Sitter, anti-de Sitter and the static Einstein universe).

## Introduction

Black hole thermodynamics is an important area of modern theoretical physics linking quantum processes and classical gravity. The thermodynamics of stationary horizons is well developed but, when horizons become dynamical (i.e., timelike or spacelike apparent/trapping horizons instead of null event horizons [1]), our understanding of their thermodynamics drops dramatically. A valuable tool to obtain the Hawking temperature of time-dependent horizons is the tunneling formalism pioneered by Parikh and Wilczek [2], which uses Painlevé–Gullstrand (PG) coordinates [3, 4] penetrating the horizon (see [5] for a review). A chacteristic feature of PG coordinates is that the three-dimensional spatial sections of spacetimes foliated by these coordinates are flat. PG coordinates constitute a very useful chart also in other problems in classical and quantum gravity where Schwarzschild-like (or “curvature”) coordinates fail [1, 6–23]. Therefore, from the point of view of tool building and in view of their many applications, it is important to have a complete understanding of PG coordinates.

It is sometimes stated explicitly in the literature that all static and spherically symmetric spacetimes admit PG coordinates, but there are such situations of physical interest where PG coordinates fail, and one must resort to less optimal tools. To be clear, we reserve the name “Painlevé–Gullstrand coordinates” for a foliation of a spherically symmetric spacetime with flat spatial sections: this is an essential feature of these coordinates that we want to preserve. Other very useful coordinates in the literature (e.g., those of [24] for the Reissner–Nordström spacetime) recast a spherically symmetric line element in a form close to the Painlevé–Gullstrand one, but do not have flat spatial sections. These situations include the Schwarzschild-anti de Sitter family of black holes. It turns out that the problem is not the black hole itself, but rather the anti-de Sitter background in which the latter is embedded. It has been pointed out that PG coordinates cannot be constructed for anti-de Sitter space [25]. On the other hand, a recipe that is quite general to construct PG coordinates in spherically symmetric spacetimes exists [12, 26]. Here we explain the difficulties with anti-de Sitter space and with many other spherical geometries (static or not), from both the mathematical and the physical points of view.

PG coordinates [3, 4] are just a special case (corresponding to a special value of the parameter) of the more general one-parameter Martel–Poisson family of charts. Therefore, we first discuss the general Martel–Poisson family. Not surprisingly, the prototypical geometry in which both PG and Martel–Poisson coordinates were originally introduced is the Schwarzschild spacetime, to which one often refers to gain physical intuition for more general situations, expecially in black hole thermodynamics. We will abide to this unwritten rule and use the Schwarzschild spacetime to shed light on different geometries.

Motivated by the puzzle with anti-de Sitter space, we consider static cosmological metrics, including de Sitter and anti-de Sitter space and the Einstein static universe. The Martel–Poisson coordinates [26] for the Schwarschild spacetime are based on radial timelike geodesics and they use as time coordinate the proper time of observers in radial free fall. Martel and Poisson [26] give a detailed mathematical construction and physical interpretation for the Schwarzschild geometry, and also outline how to construct similar coordinates for generic static spherically symmetric spacetimes [26]. In the realm of cosmology, de Sitter and anti-de Sitter spaces and the Einstein static universe are spherically symmetric and locally static, like Schwarschild. The main difference with respect to Schwarzschild in the construction of Martel–Poisson coordinates is that one needs to consider *outgoing* massive observers in radial motion starting from a centre instead of *ingoing* observers falling radially from infinity. This fact leads to some differences with respect to the Martel-Poisson treatment, which are highlighted here.

Let us begin by reviewing Martel–Poisson coordinates (and their special PG subcase) for the Schwarzschild spacetime^1^ [26]

1 $$\begin{aligned} ds^2= & {} -\left( 1-\frac{2M}{r} \right) dt^2 + \frac{dr^2}{1-2M/r} +r^2 d\varOmega _{(2)}^2 \nonumber \\\equiv & {} -fdt^2 + \frac{dr^2}{f} +r^2 d\varOmega _{(2)}^2, \end{aligned}$$

where $$d\varOmega _{(2)}^2=d\vartheta ^2 +\sin ^2 \vartheta \, d\varphi ^2$$ is the line element on the unit 2-sphere. The Martel–Poisson family of charts is parametrized by a parameter *p* spanning the range $$ 0<p\le 1$$. The family includes the Painlevé–Gullstrand coordinates (for $$p=1$$) and the Eddington–Finkelstein (EF) coordinates obtained as the limit $$p\rightarrow 0$$. Let $$\tau $$ denote the proper time along radial timelike geodesics and $$u^a $$ be the particle four-velocity, which is related to its coordinate 3-velocity by

2 $$\begin{aligned} u^{\mu }\equiv \frac{dx^{\mu }}{d\tau }= \frac{dx^{\mu }}{dt} \, \frac{dt}{d\tau } {=} \gamma \, \frac{dx^{\mu }}{dt} {=} \gamma \left( 1, \frac{d\mathbf {x}}{dt} \right) \equiv \left( \gamma , \gamma \mathbf {v} \right) ,\nonumber \\ \end{aligned}$$

where $\mathbf {v}$ is the coordinate 3-velocity and $\gamma ( v) =\left( 1-v^2\right) ^{-1/2}$ is the Lorentz factor. The Martel–Poisson coordinates for the Schwarzschild spacetime are based on *ingoing* freely falling observers with purely radial velocity. The energy *E* of a particle of mass *m* is conserved along the geodesics,

3 $$\begin{aligned} m \, u_c t^c = -E \,, \end{aligned}$$

where $t^c \equiv \left( \partial /\partial t \right) ^c$ is the timelike Killing vector. Introducing the particle energy per unit mass ${\bar{E}} \equiv E/m$, it is

4 $$\begin{aligned} \frac{dt}{d\tau } = \frac{ {\bar{E}}}{f} . \end{aligned}$$

The normalization $u_c u^c =-1$ reads

5 $$\begin{aligned} -f \left( \frac{dt}{d\tau }\right) ^2 + \frac{1}{f} \left( \frac{dr}{d\tau }\right) ^2 = -1 ; \end{aligned}$$

Equation (4) then yields

6 $$\begin{aligned} \left( \frac{dr}{d\tau }\right) ^2={\bar{E}}^2 -f \end{aligned}$$

and

7 $$\begin{aligned} \frac{dr}{d\tau } = - \gamma v = - \sqrt{ {\bar{E}}^2-f} \end{aligned}$$

with $v\equiv \left| \mathbf {v} \right|$. At $r=\infty$, the coordinate time *t* of static observers coincides with the proper time $\tau$ along the geodesics and Eq. (7) reads

8 $$\begin{aligned} v_{\infty } \equiv \left| \frac{dr}{dt} \right| _{\infty } = \sqrt{ {\bar{E}}^2-1}. \end{aligned}$$

The parameter *p* is defined as

9 $$\begin{aligned} p \equiv \frac{1}{{\bar{E}}^2} = 1-v_{\infty }^2. \end{aligned}$$

The Martel–Poisson coordinates are defined by

10 $$\begin{aligned} d{\bar{t}} = dt + \frac{\sqrt{ 1-pf}}{f}\, dr \end{aligned}$$

or, in integral form,

11 $$\begin{aligned} {\bar{t}} = t + \int \frac{\sqrt{ 1-pf}}{f}\, dr \end{aligned}$$

and the Schwarzschild line element becomes

12 $$\begin{aligned} ds^2 = -fd{\bar{t}}^2 \pm 2 \sqrt{1-pf} \, d{\bar{t}}dr +pdr^2 +r^2 d\varOmega _{(2)}^2.\nonumber \\ \end{aligned}$$

For $p=1$, which corresponds to observers infalling radially from infinity with zero initial velocity $v_{\infty }=0$, the Martel–Poisson coordinates reduce to the more familiar PG coordinates in which the Schwarzschild line element assumes the form

13 $$\begin{aligned} ds^2 = - \left( 1-\frac{2M}{r} \right) d{\bar{t}}^2 + 2 \sqrt{\frac{2M}{r}} d{\bar{t}}dr +dr^2 +r^2 \varOmega _{(2)}^2\nonumber \\ \end{aligned}$$

and

14 $$\begin{aligned} {\bar{t}} = t+4M\left( \sqrt{\frac{r}{2M} } +\ln \sqrt{ \left| \frac{\sqrt{r/(2M)} -1}{\sqrt{r/(2M)} +1} \right| } \, \right) . \end{aligned}$$

In the limit $p\rightarrow 0$, EF coordinates [28, 29] are obtained [26]. First, one introduces the tortoise coordinate

15 $$\begin{aligned} r^* \equiv r{+}2M \ln \left| \frac{r}{2M} -1\right| {=} r+2M \ln \left| \frac{r}{2M} \left( 1-\frac{2M}{r} \right) \right| ,\nonumber \\ \end{aligned}$$

whose differential satisfies

16 $$\begin{aligned} dr^* = \frac{dr}{1-2M/r}. \end{aligned}$$

The null coordinates $\left( u,v\right)$ are introduced by

17 $$\begin{aligned} du\equiv & {} dt-dr^*, \end{aligned}$$

18 $$\begin{aligned} dv\equiv & {} dt+dr^*, \end{aligned}$$

and $dt=du+dr^*=dv-dr^*$. Ingoing (−) EF coordinates [28, 29] use the advanced time *v* and the Schwarzschild line element (1) is written as

19 $$\begin{aligned} ds^2_{(-)} = -\left( 1-\frac{2M}{r}\right) dv^2 +2dvdr +r^2 d\varOmega _{(2)}^2, \end{aligned}$$

while outgoing ($+$) coordinates use the retarded time *u*, with

20 $$\begin{aligned} ds^2_{(+)} = -\left( 1-\frac{2M}{r}\right) du^2 -2dudr +r^2 d\varOmega _{(2)}^2. \end{aligned}$$

Martel and Poisson [26] proceed to generalize the construction of their coordinates to more general static and spherically symmetric spacetimes

21 $$\begin{aligned} ds^2=-\text{ e}^{-2\varPhi } fdt^2+\frac{dr^2}{f} + r^2 d\varOmega _{(2)}^2 \end{aligned}$$

with $\varPhi =\varPhi (r), f=f(r)$. By redefining the time coordinate according to

22 $$\begin{aligned} T=t+\int \frac{ \sqrt{\text{ e}^{2\varPhi } -p f}}{f} \, dr, \end{aligned}$$

the line element becomes

23 $$\begin{aligned} ds^2= & {} -\text{ e}^{-2\varPhi } f dT^2 +2\text{ e}^{-2\varPhi }\sqrt{ \text{ e}^{2\varPhi } - pf}\, dTdr \nonumber \\&+p\text{ e}^{-2\varPhi }dr^2 +r^2 d\varOmega _{(2)}^2 \end{aligned}$$

PG coordinates are obtained in the limit $p\rightarrow 1$ and 3-surfaces of constant *T* are flat [26].

This construction breaks down when the argument of the square root becomes negative. The failure for anti-de Sitter space and for the inner region of the Reissner–Nordström metric was noted in [25]. These two regions have in common a negative quasilocal mass. We show in Sect. 4 that, whenever this happens, PG coordinates cannot be introduced and we highlight the physical reason: since gravity becomes repulsive, a massive test particle with zero initial velocity cannot overcome this repulsion and it cannot even begin to travel radially along a radial timelike geodesic. For illustration, we refer to the case of the Schwarschild naked singularity (which is illuminating as usual) and then revert to anti-de Sitter space.

## Martel–Poisson family of charts for de Sitter space

Various coordinate charts in de Sitter space are reviewed in Refs. [30, 31]. Here we limit ourselves to the Poisson–Martel family of charts and its limits. Begin from the de Sitter line element in Schwarzschild-like (or curvature) coordinates

24 $$\begin{aligned} ds^2= & {} -\left( 1-H^2R^2\right) dT^2 +\frac{dR^2}{1-H^2R^2} +R^2 d\varOmega _{(2)}^2 \nonumber \\\equiv & {} -fdT^2 +\frac{dR^2}{f} +R^2 d\varOmega _{(2)}^2, \end{aligned}$$

where *H* is constant and the line element is locally static in the region $0\le R\le H^{-1}$, and define a new time coordinate ${\bar{T}}$ by^2^

25 $$\begin{aligned} d{\bar{T}}= dT + \frac{ \sqrt{1-pf}}{f} \, dR , \end{aligned}$$

where *p* is a parameter labelling different charts. Clearly, the components of the differential $d{\bar{T}}=c_1 dT +c_2 dR$ satisfy $\partial c_1 / \partial R = \partial c_2 / \partial T$ and $d{\bar{T}}$ is exact.

To see the physical meaning of *p*, write the equation of *outgoing* (${\dot{R}}>0$) radial timelike geodesics

26 $$\begin{aligned} \frac{ds^2}{d\tau ^2} = -f \left( \frac{dT}{d\tau } \right) ^2 +\frac{1}{f} \, \left( \frac{dR}{d\tau } \right) ^2 =-1, \end{aligned}$$

where $\tau$ is the proper time along timelike geodesics. Since the de Sitter metric is locally static, the energy is conserved along geodesics. If $T^a= \left( \partial /\partial T \right) ^a$ denotes the timelike Killing vector and $p^{c}=m u^c$ the four-momentum of a massive particle of mass *m* and 4-velocity $u^c$, then $p_a T^a= -E$ is constant along the geodesic. Using the energy per unit mass ${\bar{E}} \equiv E/m$, we have $u^0= dT/d\tau = {\bar{E}}/f$. Substituting into the radial timelike geodesic equation (26) yields

27 $$\begin{aligned} \left( \frac{dR}{d\tau } \right) ^2= {\bar{E}}^2-f \end{aligned}$$

and

28 $$\begin{aligned} \frac{dR}{d\tau } = \pm \sqrt{ {\bar{E}}^2-f} \end{aligned}$$

with the upper sign for outgoing and the lower sign for ingoing geodesics. Introduce the parameter $p\equiv 1/{\bar{E}}^2$ and consider the radial component of the four-velocity of the massive particle

29 $$\begin{aligned} \frac{dR}{d\tau }=\frac{dR}{dt}\, \frac{dt}{d\tau } =\pm \gamma (v) \, v =\pm \frac{v}{\sqrt{1-v^2}} =\pm \sqrt{{\bar{E}}^2-f} , \end{aligned}$$

where $\gamma (v)$ is the Lorentz factor and $v=|\mathbf {v}|$ is the magnitude of the coordinate 3-velocity.

At $R=0$ we have

30 $$\begin{aligned} \left| \frac{dR}{d\tau }\Big |_{R=0} \right| = \frac{v_0}{\sqrt{1-v_0^2}}= \sqrt{{\bar{E}}^2-1}; \end{aligned}$$

then

31 $$\begin{aligned} p\equiv \frac{1}{{\bar{E}}^2} = {1-v_0^2} . \end{aligned}$$

The range of values of the parameter *p* is $0 < p \le 1$, as for the Schwarzschild case, although now there is a difference: the observer starts out at $R=0$ instead of $R=\infty$ and it is outgoing instead of ingoing. In the de Sitter case one cannot start at $R=\infty$ because in the region $R> R_{H} \equiv H^{-1}$ beyond the de Sitter horizon the geometry is not static and there is no conserved energy *E* along timelike geodesics there.

In principle, one could consider a radially ingoing timelike observer starting out at the de Sitter horizon $$R_H $$ with velocity $$v_H \equiv -dR/d\tau \Big |_{R=H^{-1}} =-{\bar{E}}$$, but the $$\left( t, R\right) $$ coordinates fail there.

In terms of the new time coordinate ${\bar{T}}$, the line element (24) becomes

32 $$\begin{aligned} ds^2=-fd{\bar{T}}^2 \pm 2 \sqrt{1-pf} \,d{\bar{T}} dR +p dR^2 +R^2 d\varOmega _{(2)}^2 \end{aligned}$$

with the upper [lower] sign referring to ingoing [outgoing] timelike geodesics. The metric is regular at the horizon $R_H=H^{-1}$: these coordinates penetrate the horizon and the time slices $d{\bar{T}}=0$ are not flat unless $p=1$.

The relation (25) can be integrated explicitly to provide the new time

33 $$\begin{aligned} {\bar{T}}= & {} T + \int dR \, \frac{\sqrt{1-pf}}{f} \end{aligned}$$

34 $$\begin{aligned}= & {} T + \sqrt{1-p} \int dR \, \frac{ \sqrt{1+\frac{p}{1-p} \, H^2 R^2}}{ 1-H^2R^2}. \end{aligned}$$

Using ${\bar{H}} \equiv \sqrt{ \frac{p}{1-p}} \, H$ and $\alpha \equiv \sqrt{\frac{1-p}{p} } \in {\mathbb {R}}$, one obtains

35 $$\begin{aligned} {\bar{T}}= & {} T + \sqrt{1-p} \int dR \, \frac{ \sqrt{1+{\bar{H}}^2 R^2} }{1-\alpha ^2 {\bar{H}}^2R^2} \nonumber \\= & {} T + \frac{1}{\alpha ^2 {\bar{H}}} \left[ \sqrt{\alpha ^2+1} \tanh ^{-1} \left( \frac{ \sqrt{\alpha ^2+1}\, {\bar{H}}R}{ \sqrt{ 1+{\bar{H}}^2 R^2} }\right) \right. \nonumber \\&\left. -\sinh ^{-1} \left( {\bar{H}}R \right) \right] \nonumber \\= & {} T {+} \frac{\sqrt{p} }{H} \left[ \frac{1}{p} \tanh ^{-1} \left( \frac{1}{ \sqrt{p(1-p)}} \, \frac{H R }{ \sqrt{1{+} \frac{p}{1-p} \, H^2 R^2} } \right) \right. \nonumber \\&\left. -\sinh ^{-1} \left( \sqrt{ \frac{p}{1-p}}\, HR \right) \right] + \text{ const. } \end{aligned}$$

### Painlevé–Gullstrand coordinates

For the parameter value $p=1$, corresponding to vanishing initial velocity of the observer $v_0=0$, the line element (32) becomes

36 $$\begin{aligned} ds^2=-fd{\bar{T}}^2 \pm 2HR \,d{\bar{T}} dR + dR^2 +R^2 d\varOmega _{(2)}^2 \end{aligned}$$

(upper sign for ingoing, lower for outgoing geodesics), which is the de Sitter line element in Painlevé–Gullstrand coordinates, which are therefore contained in the Martel–Poisson family of charts. Now the 3-spaces of constant time ${\bar{T}}$ are Euclidean.

The Painlevé–Gullstrand time coordinate obtained from Eq. (33) for $p=1$ is

37 $$\begin{aligned} {\bar{T}}= & {} T+\int dR \, \frac{\sqrt{1-f}}{f} = T\pm \frac{1}{2H} \int dR \, \frac{HR}{1-H^2R^2} \nonumber \\= & {} T \pm \frac{1}{2H} \ln \left| 1-H^2R^2\right| +\text{ const. } \end{aligned}$$

This is precisely the coordinate called “Painlevé–de Sitter time” used to study Hawking radiation with the tunneling method in Ref. [32].

### Eddington–Finkelstein coordinates

EF coordinates for de Sitter space are used routinely, and they parallel the EF coordinates for Schwarzschild spacetime [28, 29]. The analogue of the tortoise coordinate is

38 $$\begin{aligned} R^* \equiv \frac{1}{2H} \ln \left| \frac{1+HR}{1-HR}\right| = \frac{1}{2H} \ln \left| \frac{1-H^2R^2}{\left( 1-HR\right) ^2}\right| , \end{aligned}$$

whose differential satisfies

39 $$\begin{aligned} dR^* = \frac{dR}{1-H^2R^2} . \end{aligned}$$

Null coordinates $\left( U,V\right)$ are introduced by

40 $$\begin{aligned} dU= & {} dT-dR^* , \end{aligned}$$

41 $$\begin{aligned} dV= & {} dT+dR^* , \end{aligned}$$

and

42 $$\begin{aligned} dT= & {} dU+dR^*=dV-dR^* = \frac{dU+dV}{2} ,\nonumber \\ dR^*= & {} dV-dT= dT-dU = \frac{dV-dU}{2}. \end{aligned}$$

Although the parameter *p* spans the range $\left( 0, 1 \right)$, one can formally obtain EF coordinates by taking the limit $p\rightarrow 0$, which lies outside of this range, in the relevant equations. In this limit, the line element (32) becomes

43 $$\begin{aligned} ds^2 = -\left( 1-H^2R^2\right) d{\bar{T}}^2 \pm 2d{\bar{T}}dR +R^2 d\varOmega _{(2)}^2 \end{aligned}$$

which is the well known de Sitter line element in EF coordinates ( e.g., [1, 33]), with the upper sign denoting EF coordinates based on ingoing null geodesics and the lower sign denoting those based on outgoing null geodesics. In this limit the coordinate ${\bar{T}}$ (renamed *V*) is obtained by integrating in Eq. (33):

44 $$\begin{aligned} V\equiv & {} \lim _{p\rightarrow 0} {\bar{T}} = T + \int \frac{dR}{1-H^2R^2} \nonumber \\= & {} T + \frac{1}{H} \, \tanh ^{-1} \left( HR \right) \nonumber \\= & {} T + \frac{1}{2H} \, \ln \left( \frac{1+HR}{1-HR} \right) \equiv T+R^* \end{aligned}$$

and becomes the (null) advanced time. Introducing the retarded time as the second null coordinate

45 $$\begin{aligned} U \equiv T - \frac{1}{2H} \, \ln \left( \frac{1+HR}{1-HR} \right) \equiv T-R^*, \end{aligned}$$

the line element (43) can be written as

46 $$\begin{aligned} ds^2= & {} -\left( 1-H^2R^2\right) dV\left( dV \mp 2dR^*\right) + R^2 d\varOmega _{(2)}^2 \nonumber \\= & {} -\left( 1-H^2R^2\right) \left[ dV^2 \pm dV(dV-dU )\right] \end{aligned}$$

(upper sign for ingoing and lower for outgoing geodesics), where

47 $$\begin{aligned} R\left( U,V \right) = \frac{1}{H} \, \tanh \left[ \frac{H\left( V-U \right) }{2} \right] . \end{aligned}$$

Using

48 $$\begin{aligned} dT= & {} \frac{dU+dV}{2}, \end{aligned}$$

49 $$\begin{aligned} dR= & {} \frac{\left( 1-H^2R^2\right) }{2} \left( dV-dU \right) , \end{aligned}$$

one obtains

50 $$\begin{aligned} ds_{(-)}^2 = -\left( 1-H^2R^2\right) dV\left( 2dV-dU\right) +R^2 d\varOmega _{(2)}^2 \end{aligned}$$

for ingoing null geodesics and

51 $$\begin{aligned} ds_{(+)}^2 = -\left( 1-H^2R^2\right) dUdV +R^2 d\varOmega _{(2)}^2 \end{aligned}$$

for outgoing null geodesics. These line elements can be rewritten in terms of only one null coordinate *U* or *V*, respectively, obtaining

52 $$\begin{aligned} ds^2_{(+)}= & {} -\left( 1-H^2R^2 \right) dU^2 -2dUdR +R^2 d\varOmega _{(2)}^2 \end{aligned}$$

53 $$\begin{aligned} ds^2_{(-)}= & {} -\left( 1-H^2R^2 \right) dV^2 +2dVdR +R^2 d\varOmega _{(2)}^2. \end{aligned}$$

## Martel–Poisson charts for anti-de Sitter space

Begin from the de anti-Sitter line element in curvature coordinates

54 $$\begin{aligned} ds^2= & {} -\left( 1+H^2R^2\right) dT^2 +\frac{dR^2}{1+H^2R^2} +R^2 d\varOmega _{(2)}^2 \nonumber \\\equiv & {} -fdT^2 +\frac{dR^2}{f} +R^2 d\varOmega _{(2)}^2 \end{aligned}$$

and redefine the time coordinate $T\rightarrow {\bar{T}}$ according to

55 $$\begin{aligned} d{\bar{T}}= dT + \frac{ \sqrt{1-pf}}{f} \, dR. \end{aligned}$$

The equation of outgoing radial timelike geodesics is again

56 $$\begin{aligned} \frac{ds^2}{d\tau ^2} = -f \left( \frac{dT}{d\tau } \right) ^2 +\frac{1}{f} \, \left( \frac{dR}{d\tau } \right) ^2 =-1 \end{aligned}$$

and a particle energy is conserved along geodesics, $$p_c T^c= -E $$, giving

57 $$\begin{aligned} \left( \frac{dR}{d\tau } \right) ^2= {\bar{E}}^2-f \end{aligned}$$

and

58 $$\begin{aligned} \frac{dR}{d\tau } = \sqrt{ {\bar{E}}^2-f} \end{aligned}$$

for outgoing geodesics. Introducing $p\equiv 1/{\bar{E}}^2$ and $v_0$ defined by

59 $$\begin{aligned} \frac{dR}{d\tau }\Big |_{R=0} = \sqrt{{\bar{E}}^2-1} =\gamma _0^2 v_0^2 , \end{aligned}$$

the line element (54) becomes

60 $$\begin{aligned}&ds^2=-fd{\bar{T}}^2 \pm 2 \sqrt{1-p -pH^2R^2} \,d{\bar{T}} dR\nonumber \\&\qquad \qquad +p dR^2 +R^2 d\varOmega _{(2)}^2 . \end{aligned}$$

The Martel–Poisson coordinates are only defined for

61 $$\begin{aligned} 0\le R \le \sqrt{ \frac{1-p}{p}} \, H^{-1} \equiv R_+ . \end{aligned}$$

In the limit $p\rightarrow 1^{-}$ in which one expects to recover Painlevé–Gullstrand coordinates, $R_+\rightarrow 0$ and this coordinate chart disappears.

If $0<p<1$, one can again obtain the time coordinate ${\bar{T}}$ in finite terms. Using the same ${\bar{H}}$ and $\alpha$ as in the previous section,

62 $$\begin{aligned} {\bar{T}}= & {} T+\int dR \, \frac{\sqrt{1-pf}}{f} \nonumber \\= & {} T+ \sqrt{1-p} \int dR \, \frac{ \sqrt{1-\frac{p}{1-p} \, H^2 R^2}}{ 1+H^2R^2} \nonumber \\= & {} T+ \frac{\sqrt{1-p}}{\alpha ^2 {\bar{H}}} \left[ \sqrt{\alpha ^2+1} \tan ^{-1} \left( \frac{ \sqrt{\alpha ^2+1}\, {\bar{H}}R}{ \sqrt{ 1-{\bar{H}}^2 R^2} }\right) \right. \nonumber \\&\left. -\sin ^{-1} \left( {\bar{H}}R \right) \right] \nonumber \\= & {} T {+} \frac{\sqrt{p} }{H} \left[ \frac{1}{p} \tan ^{-1} \left( \frac{1}{ \sqrt{p(1-p)}} \, \frac{H R }{\sqrt{1- \frac{p}{1{-}p} \, H^2 R^2} } \right) \right. \nonumber \\&\left. -\sin ^{-1}\left( \sqrt{ \frac{p}{1-p}}\, HR \right) \right] +\text{ const. } \end{aligned}$$

True PG coordinates for this metric, corresponding to the limit $p\rightarrow 1$, do not exist. In addition to the disappearance of the chart, ${\bar{T}}$ becomes complex in this limit. This fact was noted in Ref. [25]. We come now to the crucial point, which is more general than the anti-de Sitter geometry. For completeness, before discussing this central issue, we report the EF coordinates for anti-de Sitter space.

### Eddington–Finkelstein coordinates

One defines the tortoise coordinate $$ r^*$$ by imposing that the restriction of the metric to the 2-space $$\left( T, r^* \right) $$ is explicitly conformally flat,

63 $$\begin{aligned} -f dT^2 + f^{-1} dR^2 = f \left( -dT^2 + dr^{*2} \right) , \end{aligned}$$

hence $dr^* = dR/f = dR/\left( 1+H^2 R^2 \right)$ or, in finite form,

64 $$\begin{aligned} r^* = \int {\frac{dR}{1 + H^2 R^2}} = \frac{\tan ^{-1}{(HR)}}{H}. \end{aligned}$$

The EF retarded and advanced times $\left( u,v \right)$ are then

65 $$\begin{aligned} u\equiv & {} T - r^* = T - \frac{\tan ^{-1}{\left( HR \right) }}{H}, \end{aligned}$$

66 $$\begin{aligned} v\equiv & {} T + r^* = T + \frac{\tan ^{-1}{\left( HR \right) }}{H}. \end{aligned}$$

The outgoing and ingoing EF line elements follow by substituting $dT = du + dr^*$ and $dT = dv - dr^*$ in the line element (60),

67 $$\begin{aligned} ds_{(+)}^2= & {} -f du^2 - 2 \ du \, dR + R^2 d\varOmega _{(2)}^2, \end{aligned}$$

68 $$\begin{aligned} ds_{(-)}^2= & {} -f dv^2 + 2 \ dv \, dR + R^2 d\varOmega _{(2)}^2. \end{aligned}$$

Using $dr^* = \left( dv - du \right) /2$ in Eq. (67) yields

69 $$\begin{aligned} ds^2 = - f \ dudv + R^2 d\varOmega _{(2)}^2. \end{aligned}$$

## PG coordinates and Misner–Sharp–Hernandez mass

A rather general recipe to construct PG coordinates for any spherically symmetric metric (static or not) is given in Ref. [12]. Begin with the line element in the Abreu–Nielsen–Visser gauge [12, 13]

70 $$\begin{aligned} ds^2= & {} - \text{ e}^{2\varPhi (t,R)} \left( 1-\frac{2M_\text {MSH}(t,R)}{R} \right) dt^2 \nonumber \\&+\frac{dR^2}{ 1-2M_\text {MSH}(t,R)/ R } +R^2 d\varOmega _{(2)}^2 \end{aligned}$$

employing the areal radius *R* as the radial coordinate. Here $M_\text {MSH}(t,R)$ is the Misner–Sharp–Hernandez mass well known in spherical fluid mechanics and in gravitational collapse [34, 35]. (It is not trivial that this is the object appearing in Eq. (70) – see [12, 13] for an explanation.)

Define the new time coordinate ${\bar{t}}\left( t, R\right)$ by

71 $$\begin{aligned} d{\bar{t}} = \frac{\partial {\bar{t}}}{\partial t} \, dt + \frac{\partial {\bar{t}}}{\partial R} \, dR \equiv \dot{{\bar{t}}} dt +{\bar{t}}' dR; \end{aligned}$$

substituting into the line element and requiring $g_{RR}=1$ leads to [12]

72 $$\begin{aligned} {\bar{t}}'=\pm \frac{ \sqrt{ 2M_\text {MSH}/R}}{1-2M/R} \, \text{ e}^{\varPhi } \, \dot{{\bar{t}}} , \end{aligned}$$

which has always a solution. Then the line element in PG coordinates takes the form

73 $$\begin{aligned} ds^2= & {} - \left[ c^2\left( {\bar{t}}, R \right) -v^2\left( {\bar{t}}, R \right) \right] d{\bar{t}}^2 + 2 v\left( {\bar{t}}, R \right) d{\bar{t}}dR \nonumber \\&+R^2 d\varOmega _{(2)}^2 , \end{aligned}$$

where

74 $$\begin{aligned} c\left( {\bar{t}}, R \right)= & {} \frac{ \text{ e}^{-\varPhi } }{\dot{{\bar{t}}}} \,, \end{aligned}$$

75 $$\begin{aligned} v \left( {\bar{t}}, R \right)= & {} c\left( {\bar{t}}, R \right) \, \sqrt{ \frac{2M_\text {MSH}}{R} } \le c. \end{aligned}$$

In practice, the function ${\bar{t}}\left( t, R \right)$ is not always determined explicitly. This is equivalent to introducing an integrating factor to make $d{\bar{t}}$ an exact differential [1].

It is clear that the Nielsen–Visser procedure breaks down in regions where the mass $M_\text {MSH}$ becomes negative and *v* becomes imaginary. This is exactly the case of anti-de Sitter space in the region $0\le R<H^{-1}$ covered by the locally static coordinates, and of the inner region of the Reissner–Nordström spacetime pointed out in [25] (although the procedure of [12] to construct PG coordinates is not mentioned there). Trivial as it may seem, this observation explains from the *mathematical* point of view why one cannot construct PG coordinates in these two spaces and, more in general, in any region with negative Misner–Sharp–Hernandez mass.

Let us come now to the *physical* explanation. As usual, the Schwarzschild spacetime taken as an example sheds light on other geometries. Consider the Schwarzschild spacetime (1) with negative mass, which has a naked central singularity and no horizons. The Misner-Sharp-Hernandez mass is $M_\text {MSH}=-|m|<0$ and PG coordinates cannot be constructed. The reason is that these coordinates are associated with observers falling in radially from infinity with zero initial velocity. Since gravity is now repulsive, these particular observers cannot even begin to fall because they cannot overcome the repulsion and must move outwards instead. There are no ingoing timelike radial geodesics with zero initial velocity. To wit, repeat the procedure of Sect. 1 to obtain, along radial timelike geodesics,

76 $$\begin{aligned} \left( \frac{dr}{d\tau } \right) ^2 ={\bar{E}}^2-f={\bar{E}}^2 -1 -\frac{2|M|}{r}; \end{aligned}$$

imposing zero initial velocity at infinity gives

77 $$\begin{aligned} v_{\infty }^2 = \left( \frac{dr}{d\tau } \right) ^2 \Big |_{\infty }= {\bar{E}}^2-1=0 \end{aligned}$$

or ${\bar{E}}=1$. Then at any radius $r\in \left( 0, +\infty \right)$ it is

78 $$\begin{aligned} \left( \frac{dr}{d\tau } \right) ^2 = -\frac{2|M|}{r} <0 , \end{aligned}$$

whic is clearly impossible. Therefore, PG observers cannot be defined because of the repulsion. The situation is the same in anti-de Sitter space, except that now the observer starts at the centre. We have again (changing $f \rightarrow 1+H^2R^2$),

79 $$\begin{aligned} \left( \frac{dr}{d\tau } \right) ^2 = {\bar{E}}^2-f = {\bar{E}}^2-1-H^2R^2 \end{aligned}$$

and, imposing that the initial velocity at the centre vanishes,

80 $$\begin{aligned} v_0^2 \equiv \left( \frac{dR}{d\tau } \right) ^2 \Big |_{R=0} ={\bar{E}}^2-1=0 , \end{aligned}$$

one obtains again ${\bar{E}}=1$ and

81 $$\begin{aligned} \left( \frac{dr}{d\tau } \right) ^2 =-H^2R^2 <0 \end{aligned}$$

for all $R\in \left( 0, H^{-1} \right)$, which clearly shows the impossibility of defining PG observers. This is due to the fact that the negative cosmological constant repels and confines a particle at the centre. If the particle has zero initial velocity there, it will not exit. By contrast, the positive cosmological constant of de Sitter space attracts a particle located at $R=0$ toward larger and larger values of *R*.

## Einstein static universe

In general relativity, the static Einstein universe [36] arises from the delicate balance between a dust and the positive cosmological constant, and is unstable with respect to homogenous perturbations [37]. Stability with respect to vector and tensor perturbations is a different issue, and stability with respect to inhomogeneous scalar density perturbations depends on the sound speed $c_s$ [38–40], with neutral stability occurring if $c_s>1/\sqrt{5}$, a range that also maximizes entropy [39].

Modern interest in this solution arises in braneworld models [41–44], loop quantum cosmology [45, 46], string theory [47], analog gravity [48], with generalizations to non-constant pressure [49–53]. Further motivation for the study of the static Einstein universe comes from the possibility that the early inflationary universe might have begun in an asymptotic Einstein state [54, 55]. Moreover, the static Einstein universe has seen renewed attention as a solution of the field equations of modified gravity theory [56–63]. Our considerations in this section are independent of the theory of gravity.

For the positively curved Einstein static universe, introducing the Martel-Poisson coordinates proceeds as outlined in [26]. The line element is

82 $$\begin{aligned} ds^2=-dt^2+a_0^2 \left( \frac{dr^2}{1-r^2} +r^2 d\varOmega _{(2)}^2 \right) \,, \end{aligned}$$

where $0\le r<1$ and $a_0$ is constant. This geometry has the timelike Killing vector $t^a = \left( \partial /\partial t \right) ^a$ and areal radius $R=a_0r$. The energy *E* of a test particle is conserved along the geodesic. Along radial timelike geodesics, $dt/d\tau ={\bar{E}} \equiv E/m$ and, substituting into the normalization $u_c u^c=-1$ yields

83 $$\begin{aligned} \left( \frac{dr}{d\tau }\right) ^2 = \frac{\left( {\bar{E}}^2-1 \right) }{a_0^2}\, \left( 1-r^2 \right) . \end{aligned}$$

The proper 3-velocity at $r=0$ has magnitude

84 $$\begin{aligned} v_0=\left| \frac{dr}{d\tau } \right| = \frac{ \sqrt{{\bar{E}}^2-1}}{a_0} \end{aligned}$$

so that the parameter *p* is again

85 $$\begin{aligned} p \equiv \frac{1}{{\bar{E}}^2} = 1-v_0^2, \end{aligned}$$

it has the same meaning as in the de Sitter universe, and it spans the range $0< p \le 1$. Defining the new time coordinate ${\bar{t}}$ by [26]

86 $$\begin{aligned} d{\bar{t}}=dt + \sqrt{ \frac{1-p}{1-r^2} } \, dR , \end{aligned}$$

the line element becomes

87 $$\begin{aligned} ds^2 = -d{\bar{t}}^2 +2\sqrt{ \frac{1-p}{1-r^2} } \, d{\bar{t}} dR +\frac{ pdR^2}{1-r^2} +R^2 d\varOmega _{(2)}^2 .\nonumber \\ \end{aligned}$$

Using $\alpha \equiv \sqrt{ (1-p)/p}$, the integration of Eq. (86) gives

88 $$\begin{aligned} {\bar{t}}= & {} t + a_0 \sqrt{1-p} \int \frac{dr}{ \sqrt{1 - r^2} } \nonumber \\= & {} t+ a_0 \sqrt{1-p} \arcsin r + \text{ const. }\nonumber \\\equiv & {} t+ a_0 \sqrt{1-p} \,\, \chi + \text{ const. }, \end{aligned}$$

where $\chi$ is the usual hyperspherical radius [27]. The proper radius $a_0\chi$ (which could also be called “volume” radius) is distinct from the areal radius *R* in spatially curved FLRW universes.

### PG coordinates

By taking the limit $p\rightarrow 1$, $d{\bar{t}}$ reduces to *dt* in Eq. (86), and the line element (87) reverts to the static FLRW line element (82) in comoving coordinates, in which the spatial sections are positively curved. Again, setting $v_0=0$ implies ${\bar{E}}=1$ and $\left( dr/d\tau \right) ^2 <0$ along radial timelike geodesics. PG coordinates cannot be introduced as a limit of the Poisson-Martel family of charts. The reason is rather simple: since the matter content of this universe is dust and its collapse is (just) balanced by the positive cosmological constant, a test particle with zero radial initial velocity, i.e., initially comoving with the cosmic substratum, remains comoving with it – that is, not moving at all. Massive particles on timelike radial geodesics need nonzero initial velocity to move.

PG coordinates can still be introduced following the procedure of [12, 13], which yields $M_\text {MSH}(R)=R^3/\left( 2a_0^2\right)$ and

89 $$\begin{aligned} c\left( {\bar{t}},R \right)= & {} \frac{a_0}{R} \, {\bar{t}}' , \end{aligned}$$

90 $$\begin{aligned} v\left( {\bar{t}},R \right)= & {} \pm \frac{2a_0}{R} \, {\bar{t}}' , \end{aligned}$$

and the line element in PG coordinates is

91 $$\begin{aligned} ds^2{=}-\frac{a_0^2}{R^2} ({\bar{t}}')^2 \left( 1-\frac{R}{a_0} \right) d{\bar{t}}^2 \pm \frac{2a_0 {\bar{t}}'}{R}\, d{\bar{t}}dR {+} R^2 d\varOmega _{(2)}^2 .\nonumber \\ \end{aligned}$$

The Martel–Poisson interpretation of PG coordinates does not apply to the static Einstein universe.

### Eddington–Finkelstein coordinates

The tortoise coordinate $r^*$ is defined so that $dr^* = dR/\sqrt{ f}$ and, integrating,

92 $$\begin{aligned} r^* = \int {\frac{dR}{\sqrt{1-\left( \frac{R}{a_0} \right) ^2}}} = a_0 \sin ^{-1}{r} = a_0 \chi , \end{aligned}$$

where $\chi$ is the usual hyperspherical radius [27]. The retarded and advanced times are now

93 $$\begin{aligned} u&\equiv t - r^* = t - a_0 \sin ^{-1}{r} , \end{aligned}$$

94 $$\begin{aligned} v&\equiv t + r^* = t + a_0 \sin ^{-1}{r} . \end{aligned}$$

As expected, $$v=\lim _{p\rightarrow 0} {\bar{t}}$$.

With the substitutions $dt = du + dr^*$ and $dt = dv - dr^*$, the outgoing/ingoing EF line elements are

95 $$\begin{aligned} ds_{(+)}^2&= -du^2 - \frac{2 dudR}{\sqrt{1 - r^2}}+ R^2 d\varOmega _{(2)}^2, \end{aligned}$$

96 $$\begin{aligned} ds_{(-)}^2&= -dv^2 + \frac{2 dvdR}{\sqrt{1 - r^2}} + R^2 d\varOmega _{(2)}^2 . \end{aligned}$$

Then, using $dr^* = \left( dv - du\right) /2$, one obtains

97 $$\begin{aligned} ds^2&= -dudv + R^2 d\varOmega _{(2)}^2 . \end{aligned}$$

## Conclusions

The PG coordinates originally introduced for the Schwarzschild geometry [3, 4] have proved very useful in the study of the thermodynamics of black holes and other horizons, especially in the context of the tunneling formalism of Parikh and Wilczek [2, 32]. It is rather unfortunate that this coordinate chart cannot be introduced for the most important space of string theories, anti-de Sitter space associated with a negative cosmological constant, and for the Schwarzschild-anti de Sitter geometry obtained by embedding the Schwarzschild black hole into it. This difficulty has been noted, but not explained, in the literature and its physical intepretation has remained a puzzle. One can approach the problem geometrically by attempting to foliate a static spherical spacetime with a flat foliation ( e.g., [21, 25]), but this avenue does not offer physical insight. We have clarified the anomaly by looking at the physical meaning of PG observers in static cosmological spacetimes. While, in asymptotically flat spherical spacetimes, PG observers fall in radially from infinity, starting with zero initial velocity, in cosmological settings instead they fall outward from $R=0$. In anti-de Sitter space, where the Misner–Sharp–Hernandez quasilocal mass is negative and repulsive because of the negative cosmological constant, a would-be PG observer starting at the centre with zero initial velocity cannot overcome this repulsion and move away. Similarly, in the Schwarzschild spacetime with negative mass, an observer located at infinity with zero initial velocity does not fall radially toward smaller radii because it is repelled by the negative mass at the central singularity.

This physical interpretation applies to generic regions containing negative Misner–Sharp–Hernandez mass, which repels instead of attracting. Martel-Poisson observers different from PG ones, and Lorentz-boosted with respect to them, start out radially with non-vanishing initial velocity and have a chance to overcome the initial repulsion, at least for part of their journey before they are turned around by repulsion, which causes Martel–Poisson coordinates to have a range smaller than the entire locally static region (cf. Eq. (61) for anti-de Sitter space).

In the case of (non-extremal) Schwarzschild-(anti)-de Sitter black holes, where there are two horizons, radial timelike geodesics cannot start ar $R=0$ nor at $R=\infty$. In this case it is more convenient to drop an observer somewhere in between [64], but then the physical meaning of the Martel–Poisson observers (and associated coordinates) is altered. This situation will be discussed separately.

## Data Availability

This manuscript has no associated data or the data will not be deposited. [Authors’ comment: There are no data associated to this work because of its theoretical nature.]

## Footnotes

1 We follow the notation of Ref. [27].

2 Changing the sign of the term in *dR* in Eq. (25) leaves $d{\bar{T}}$ an exact differential and changes the sign of $dR/d\tau$ and switches from ingoing to outgoing geodesics and vice-versa. Since in the following we are already considering both outgoing and ingoing geodesics, we do not need to include a ± sign here.
